# Comparison of two doses of hypobaric bupivacaine in unilateral spinal anesthesia for hip fracture surgery: 5 mg versus 7.5 mg

**DOI:** 10.11604/pamj.2017.28.108.11421

**Published:** 2017-10-04

**Authors:** Mohamed Kahloul, Mohamed Said Nakhli, Amine Chouchene, Nidhal Chebbi, Salah Mhamdi, Walid Naija

**Affiliations:** 1 Department of Anesthesia and Intensive Care, Sahloul Teaching Hospital; Faculty of Medicine “Ibn El Jazzar”, Sousse, Tunisia

**Keywords:** Unilateral spinal anesthesia, hypobaric bupivacaine, low doses, hip fracture

## Abstract

**Introduction:**

Hip fracture is a frequent and severe disease. Its prognosis depends on the perioperative hemodynamic stability which can be preserved by the unilateral spinal anesthesia especially with low doses of local anesthetics. This study aims to compare the efficacy and hemodynamic stability of two doses of hypobaric bupivacaine (7.5 mg vs 5 mg) in unilateral spinal anesthesia.

**Methods:**

In this prospective, randomized, double-blind study, 108 patients scheduled for hip fracture surgery under unilateral spinal anesthesia were enrolled to receive either 5 mg (group 1) or 7.5 mg (group 2) of hypobaric bupivacaine. Spinal anesthesia was performed in lateral position. Patients’ socio-demographic characteristics, hemodynamic profile, sensory and motor blocks parameters were recorded.

**Results:**

Both groups were comparable regarding to demographic data. Two cases of failure occurred in group 1 and one case in group 2 corresponding to a comparable efficiency rates (96.29% and 98.14% respectively; *p* = 0.5). A higher mean onset and lower mean regression times of sensory block were significantly noted in group 1 (7.79±3.76 min vs 5.75±2.35 min, p < 0.001 and 91.29±31.55 min vs 112.77±18.77 min, p <0.001 respectively). Incidence of bilateralization (29.62% vs 87.03%, p < 0.001), incidence of hypotensive episodes (59.25% vs 92.59%, p < 0.001) and vascular loading (1481.48±411.65 ml vs 2111.11±596.10 ml, p < 0.001) were significantly higher in group 2.

**Conclusion:**

The dosage of 5mg of hypobaric bupivacaine in unilateral spinal anesthesia is as effective as the dosage of 7.5 mg with lower bilateralization incidence and better hemodynamic stability.

## Introduction

Hip fracture is a frequent and severe disease that affects mainly old patients with comorbid conditions [1, 2]. It represents a major problem of public health because of its high incidence and morbi-mortality [1-4]. The prognosis of this disease depends on the comorbidities and the quality of perioperative care [1-7]. Indeed, any support delay worsens the patient outcome [5-7]. In addition, the choice of anesthetic technique is essential as it interferes with the perioperative hemodynamic status and the postoperative rehabilitation quality [8-10]. The high incidence of coronary diseases in patients with proximal femur fracture makes them more vulnerable to hypotensive episodes with increased risk of perioperative myocardial ischemia [11]. Overall, both general and regional anesthesia are possible but spinal anesthesia is the most used technique [8, 9, 12]. The reduced cardiovascular compensation mechanisms in the elderly increase significantly the frequency and severity of hypotensive episodes by sympathetic block in spinal anesthesia [13]. However, despite a better intraoperative hemodynamic stability with general anesthesia, several published studies are rather in favor of regional anesthesia [8, 9, 12]. In fact, spinal anesthesia may also provide satisfactory hemodynamic stability via sympathetic block reduction. Several solutions have been proposed, such as continuous spinal anesthesia (CSA) and even better, unilateral spinal anesthesia (ULSA) [14-17] especially when low doses of local anesthetic are used [18-20]. This study aims to compare the efficacy and safety of two doses of hypobaric bupivacaine (7.5 mg vs 5 mg) in unilateral spinal anesthesia.

## Methods

This is a prospective, randomized, double-blind study, conducted in the orthopedic surgery operating room of Sahloul teaching hospital (Sousse, Tunisia) during a 9-month period. All patients scheduled for hip fracture surgery under ULSA, were included in the study after their consent. Exclusion criteria were the contraindications of spinal anesthesia, a BMI > 30, diabetes mellitus at the stage of peripheral neuropathy, uncooperative patients and those proposed for total hip replacement.

After the approval of the ethics committee, patients were randomized into two groups depending on the dose of hypobaric bupivacaine used in the ULSA: group 1 (5 mg) and group 2 (7.5 mg). Randomization was done according to a pre-established form. Both preparations had the same volume (3 ml) and were carried out by a member of the anesthetic team not involved in patients’ therapeutic management. For group 1, the preparation contained 1 ml of isobaric bupivacaine 0.5%, 1.5 ml of distilled water and 25 μg of Fentanyl. For group 2, the preparation contained 1.5 ml of isobaric bupivacaine 0.5%, 1 ml of distilled water and 25 μg of Fentanyl. The sample size was estimated at 54 patients per group after assuming a difference in therapeutic effectiveness between the two groups not exceeding 10% and considering an α risk at 5% and a β risk at 20%.

In the operating room, the patient was supine with standard monitoring (heart rate, noninvasive blood pressure and SpO2). After premedication with 0.05 mg/kg of midazolam, an ilio-fascial block was performed with 20 ml of lidocaine epinephrine 1%. Then, the patient was placed in lateral position, limb to operate above, while maintaining the table in neutral position. Spinal anesthesia was performed in the L3-L4 or L4-L5 spaces. The end of the injection of the local anesthetic marked the reference time (T0).

A systematic loading with 5 ml/kg of isotonic saline was performed. The patient was kept in slightly downhill lateral decubitus for 15 minutes and then transferred on operating table. The collected parameters were socio-demographic characteristics of patients; hemodynamic parameters; the sensory block assessed by the pinprick test (onset time, duration and level); the motor block assessed by a two levels scale (present or absent). The primary endpoint was the efficacy of the ULSA. The secondary endpoints were the arterial blood hypotension, the ephedrine and fluid resuscitation requirements, patient and surgeon satisfaction. ULSA failure was defined as a sensory level block in the dependent limb not reaching D12 at 15 minutes and imposing in this case general anesthesia conversion. A hypotension was defined as a decrease in systolic blood pressure ≥ 20% from baseline. It was treated with iterative boli of 6 mg of Ephedrine and vascular loading with 7 ml / kg of Voluven *(HES 130 / 0.4). Statistical analysis was performed with the version 18.00 of SPSS software. Continuous data were analyzed with the Anova test. Categorical data were analyzed with the chi-2 test. A p value < 0.05 was considered statistically significant.

## Results

Our study included 108 patients. The mean age was 80.73 ± 7.64 years (with extremes ranging from 65 to 96 years). The sex ratio was 1.2. The mean BMI was 24.12 kg / m^2^ ± 3.04 (with extremes ranging from 17.78 to 29.89). ASA score ≥ 3 was found in 18 cases corresponding to 16.7% of the population. The two study groups were found to be similar regarding to demographic data (Table 1). The mean duration of surgery was also comparable in both groups (59.40 ± 26.69 min *vs* 57.77 ± 17.61 min; p = 0.77). Three cases of failure were noted in the study. Two cases occurred in group 1 corresponding to an efficiency rate of 96.29%. One case occurred in group 2 corresponding to an efficiency rate of 98.14%. Thus, the efficacy was comparable in both groups (*p* = 0.5).

**Table 1:** Demographic characteristics of the two studied groups

	Group 1n=54	Group 2n=54	*p* value
Age (years)	80.57 ± 8.52	80.88 ± 6.73	0.29
Sex ratio	0.86	1.7	0.061
BMI (kg/m²)	24.42 ± 2.94	23.99 ± 2.85	0.63
ASA status ≥ 3	49 (90.7%)	41 (75.92%)	0.076
**Treatment**			
Converting enzyme inhibitor	19 (35.18%)	23 (42.59%)	0.55
Beta blocker	7 (12.96%)	11 (20.37%)	0.22
Diuretics	2 (3.70 %)	1 (1.85%)	0.5

BMI: body mass index, ASA: American Society of Anesthesiologists.

**Figure 1:**
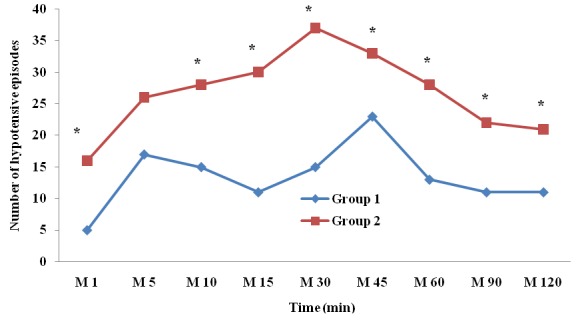
Patients’ hemodynamic profile depending on the anesthetic technique (*: p < 0.05)

The mean onset time of sensory block was significantly higher in group 1 (7.79 ± 3.76 min *vs* 5.75 ± 2.35 min; p < 0.001). The mean time of regression of sensory block was significantly lower in group 1 (91.29 ± 31.55 min *vs* 112.77 ± 18.77 min; p < 0.001). The incidence of bilateralization was significantly higher in group 2 (Table 2). Aside from the fifth minute, the incidence of hypotensive episodes was significantly higher in group 2 (Figure 1). The vascular loading was also significantly higher in group 2 (1481.48 ± 411.65 ml *vs* 2111.1 ± 596.10 ml; p < 0.001). Ephedrine consumption was higher in group 2 with no significant difference (Table 2). Patient satisfaction was better in group 1 while that of surgeons were better in group 2 with no significant difference (Table 2).

**Table 2:** Anesthetics patient characteristics

	Group 1 n=54	Group 2 n=54	*p* value
**Sensory block**			
Mean onset time (min)	7.79 ± 3.76	5.75 ± 2.35	< 0.001
Mean duration (min)	91.29 ± 31.55	112.77 ± 18.77	< 0.001
**Motor block**			0.18
present	46 (85.18 %)	50 (92.59 %)	
absent	8 (14.81 %)	4 (7.40 %)	
Bilateralization block n (%)	16 (29.62 %)	47 (87.03 %)	0.0001
Failure of unilateral spinal anesthesia	2 (3.70 %)	1 (1.85 %)	0.5
At least one hypotensive episode	32 (59.25%)	50 (92.59 %)	< 0.001
Average ephedrine consumption (mg)	7.61 ± 7.63	23.88 ± 10.28	0.53
Average need of intravenous fluids (ml)	1481.48 ± 411.65	2111.11 ± 596.10	< 0.001
**Satisfaction n (%)**			
patient	50 (92.59 %)	48 (88.88 %)	0.37
surgeon	50 (92.59 %)	53 (98.14 %)	0.18

## Discussion

In our study, we compared the dose of 5 mg hypobaric bupivacaine in the ULSA to the dose of 7.5 mg. The results showed a comparable efficiency rate in both groups with significant lower incidence of hypotensive episodes in group 1. The feasibility of spinal anesthesia with low doses of local anesthetic has been widely studied in the literature but mainly for conventional spinal anesthesia with hyperbaric bupivacaine.

Nair et al published a literature review analyzing clinical studies of spinal anesthesia for arthroscopic knee surgery. Fifteen randomized controlled trials were included with 1248 patients in total. Among these studies, five trials (387 patients) compared the effectiveness of several hyperbaric bupivacaine doses ranging from 3 to15 mg. The authors concluded that the dose of 4 to 5 mg was sufficient [21].

The effectiveness of such low doses was also found by Minville et al in a study of 74 patients aged over 75 years and scheduled for hip fracture surgery under spinal anesthesia with isobaric bupivacaine. The patients were divided into two groups: a 7.5 mg conventional spinal anesthesia group and CSA group using iterative boli of 2.5 mg. The mean bupivacaine requirement in the later group was 5 mg which were sufficient to ensure suitable surgical conditions. In addition, it had allowed a better hemodynamic stability. In fact, the occurrence of hypotensive episodes and ephedrine consumption were significantly lower in the CSA group [19].

These low doses were subsequently tested with the ULSA. Kaya et al evaluated the efficacy of 7.5 mg of bupivacaine, in 50 patients scheduled for lower limb orthopedic surgery and divided into two groups. A hyperbaric bupivacaine group was compared to a hypobaric bupivacaine group. Both protocols were effective with intraoperative hemodynamic stability, rapid regression of sensory and motor block, at the expense of a higher risk of bilateralization in hypobaric bupivacaine group [22]. The efficacy of such dose in ULSA was also confirmed by Khatouf et al in an observational study of 25 patients aged over 80 years and operated for proximal femur fracture [23].

Imbelloni et al tested the 5 mg dose of bupivacaine in ULSA in a study of 150 patients scheduled for unilateral orthopedic surgery. The patients were divided into three groups of 50 patients each according to the local anesthetic baricity (hypobaric, isobaric or hyperbaric). The three protocols were effective. However, the risk of bilateralization was more important in the isobaric group. The level and the mean duration of the sensitive blockade were significantly higher in the hypobaric group [24].

Kiran et al conducted a study on 40 patients scheduled for outpatient knee arthroscopy under hyperbaric bupivacaine ULSA. Patients were divided into 2 groups of 3 and 4 mg. The authors concluded to the efficacy of 3 mg [25]. Local anesthetics doses have been reduced by the addition of opioids [26-28]. The synergistic effect of opioids has been widely studied in the literature. Maves et al, in an animal study of 24 rats confirmed the synergistic effect of intrathecal morphine-lidocaine combination on nociception [26]. Tejwani et al have also studied this synergistic effect on rats. They explained it by conformational changes in spinal opioid receptors (delta and kappa) induced by bupivacaine and resulting in an increase of the affinity of these morphine receptors [27]. Wang et al have shown that this synergy affects only nociceptive afferent pathways and spares sympathetic efferent ones [28].

Thereby, opioids have reduced local anesthetics doses in spinal anesthesia, and therefore, the importance of sympathetic block which is dose-dependent, without having specific effects on sympathetic efferent pathways [24-28]. These two mechanisms largely explain the hemodynamic stability with low doses of local anesthetics. This justifies the interest of seeking the lowest possible dose in order to better preserve patient hemodynamic status. The same objective also justifies the use of ULSA, since the unilateral distribution of local anesthetic contributes to the reduction of sympathetic block [15, 22, 24, 29]. Kaya et al found lesser risk of bilateralization with hyperbaric mixture [22]. However, better results were found with hypobaric mixture in the trial of Imbelloni et al [24].

The peroperative hemodynamic instability is related to cardiovascular repercussions of neuraxial blockade and patient compensation mechanisms which are often altered by aging and comorbidities [11]. Its prevention is based essentially on limiting the cardiovascular impact of neuraxial blockade. At least two means are easy and practical: limitation of local anesthetic doses and one-siding sympathetic block. Several studies have confirmed the effectiveness of these techniques either associated or separated. Thus, the risk of hemodynamic instability ranging from 25 to 69% with conventional spinal anesthesia using ordinary doses decreases significantly with the ULSA in particular with low dosage [15, 30, 31].

In our study, the probability of having at least one hypotensive episode under hypobaric bupivacaine ULSA was 59.25 % in the 5 mg group versus 92.59 % in the 7.5 mg group. Thus, the later dosage is associated with 8.59 times more likely to have hypotensive episodes that the first one. In addition, vascular loading requirements (2111.11 ± 596.10 ml versus 1481.48 ± 411.65 ml; p < 0.001) and sympathomimetic consumption (23.89 ± 10.28 mg versus 7.61 ± 7.63 mg; p =0.53) were lower in the 5 mg group.

While most studies agree about the feasibility of conventional or ULSA with low doses of local anesthetic, discrepancies exist regarding onset and regression times of sensory block. Both timeframes should be interpreted with caution before mistakenly conclude to spinal anesthesia failure. Compared to conventional spinal anesthesia with usual doses, the onset time is extended ranging from 15 to 30 minutes in the literature, whereas the regression period is shorter ranging from 30 to 140 min [21, 24, 32, 33]. These thresholds must be interpreted according to the dosage of used opioid and the considered judgment criteria.

In the end, our study which is the first to compare two low doses of hypobaric bupivacaine in ULSA, has some limitations that must be considered in interpreting the results. First, the two doses used had not the same baricity. Second, the risk of bilateralization, interfering with hemodynamic stability regardless of the administered dose, has not been well studied in particular concerning its predictive factors. Third, the time chosen to declare the failure of the ULSA (15 minutes) was short since the onset time can take up to 30 minutes.

## Conclusion

The dosage of 5mg of hypobaric bupivacaine in unilateral spinal anesthesia is as effective as the dosage of 7.5 mg with lower bilateralization incidence and better hemodynamic stability.

### What is known about this topic

- Spinal anesthesia is known to induce frequent and severe hypotensive episodes mostly in the elderly;
- Unilateral spinal anesthesia may also provide satisfactory hemodynamic stability via sympathetic block reduction especially with low doses of bupivacaine.

### What this study adds

- The dosage of 5 mg of hypobaric bupivacaine in unilateral spinal anesthesia is as effective as the dosage of 7.5 mg;
- Low dose of hypobaric bupivacaine in unilateral spinal anesthesia can also be compared to new short-acting local anesthetics which are not currently available in many countries.

## Competing interests

The authors declare no competing interests.
